# Maternal and neonatal outcomes according to the timing of diagnosis of hyperglycaemia in pregnancy: a nationwide cross-sectional study of 695,912 deliveries in France in 2018

**DOI:** 10.1007/s00125-023-06066-4

**Published:** 2024-01-05

**Authors:** Nolwenn Regnault, Elodie Lebreton, Luveon Tang, Sandrine Fosse-Edorh, Yaya Barry, Valérie Olié, Cécile Billionnet, Alain Weill, Anne Vambergue, Emmanuel Cosson

**Affiliations:** 1 Santé Publique France, the national public health agency, Saint-Maurice, France; 2 French National Health Insurance (CNAM), Paris, France; 3 EPI-PHARE Epidemiology of Health Products, French National Agency for Medicines and Health Products Safety (ANSM) and French National Health Insurance (CNAM), Saint-Denis, France; 4 Endocrinology, Diabetology, Metabolism and Nutrition Department, Lille University Hospital, European Genomics Institute for Diabetes, University of Lille, Lille, France; 5 Department of Diabetology-Endocrinology-Nutrition, CRNH-IdF, CINFO, Paris 13 University, Sorbonne Paris Cité, AP-HP, Avicenne Hospital, Bobigny, France; 6 Nutritional Epidemiology Research Team (EREN), Center of Research in Epidemiology and StatisticS (CRESS), Université Sorbonne Paris Nord and Université Paris CitéInserm, INRAE, CNAM, Bobigny, France

**Keywords:** Caesarean section, Diabetes, Gestational diabetes mellitus, Macrosomia, Overt diabetes in pregnancy, Perinatal death, Preeclampsia, Pregnancy, Prematurity

## Abstract

**Aims/hypothesis:**

We aimed to assess maternal–fetal outcomes according to various subtypes of hyperglycaemia in pregnancy.

**Methods:**

We used data from the French National Health Data System (Système National des Données de Santé), which links individual data from the hospital discharge database and the French National Health Insurance information system. We included all deliveries after 22 gestational weeks (GW) in women without pre-existing diabetes recorded in 2018. Women with hyperglycaemia were classified as having overt diabetes in pregnancy or gestational diabetes mellitus (GDM), then categorised into three subgroups according to their gestational age at the time of GDM diagnosis: before 22 GW (GDM_<22_); between 22 and 30 GW (GDM_22–30_); and after 30 GW (GDM_>30_). Adjusted prevalence ratios (95% CI) for the outcomes were estimated after adjusting for maternal age, gestational age and socioeconomic status. Due to the multiple tests, we considered an association to be statistically significant according to the Holm–Bonferroni procedure. To take into account the potential immortal time bias, we performed analyses on deliveries at ≥31 GW and deliveries at ≥37 GW.

**Results:**

The study population of 695,912 women who gave birth in 2018 included 84,705 women (12.2%) with hyperglycaemia in pregnancy: overt diabetes in pregnancy, 0.4%; GDM_<22_, 36.8%; GDM_22–30_, 52.4%; and GDM_>30_, 10.4%. The following outcomes were statistically significant after Holm–Bonferroni adjustment for deliveries at ≥31 GW using GDM_22–30_ as the reference. Caesarean sections (1.54 [1.39, 1.72]), large-for-gestational-age (LGA) infants (2.00 [1.72, 2.32]), Erb’s palsy or clavicle fracture (6.38 [2.42, 16.8]), preterm birth (1.84 [1.41, 2.40]) and neonatal hypoglycaemia (1.98 [1.39, 2.83]) were more frequent in women with overt diabetes. Similarly, LGA infants (1.10 [1.06, 1.14]) and Erb’s palsy or clavicle fracture (1.55 [1.22, 1.99]) were more frequent in GDM_<22_. LGA infants (1.44 [1.37, 1.52]) were more frequent in GDM_>30_. Finally, women without hyperglycaemia in pregnancy were less likely to have preeclampsia or eclampsia (0.74 [0.69, 0.79]), Caesarean section (0.80 [0.79, 0.82]), pregnancy and postpartum haemorrhage (0.93 [0.89, 0.96]), LGA neonate (0.67 [0.65, 0.69]), premature neonate (0.80 [0.77, 0.83]) and neonate with neonatal hypoglycaemia (0.73 [0.66, 0.82]). Overall, the results were similar for deliveries at ≥37 GW. Although the estimation of the adjusted prevalence ratio of perinatal death was five times higher (5.06 [1.87, 13.7]) for women with overt diabetes, this result was non-significant after Holm–Bonferroni adjustment.

**Conclusions/interpretation:**

Compared with GDM_22–30_, overt diabetes, GDM_<22_ and, to a lesser extent, GDM_>30_ were associated with poorer maternal–fetal outcomes.

**Graphical Abstract:**

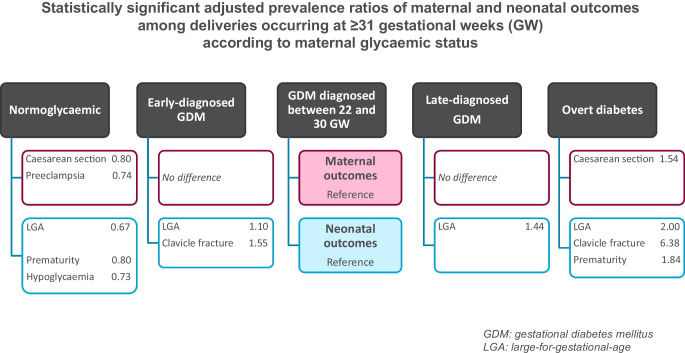

**Supplementary Information:**

The online version contains peer-reviewed but unedited supplementary material available at 10.1007/s00125-023-06066-4.


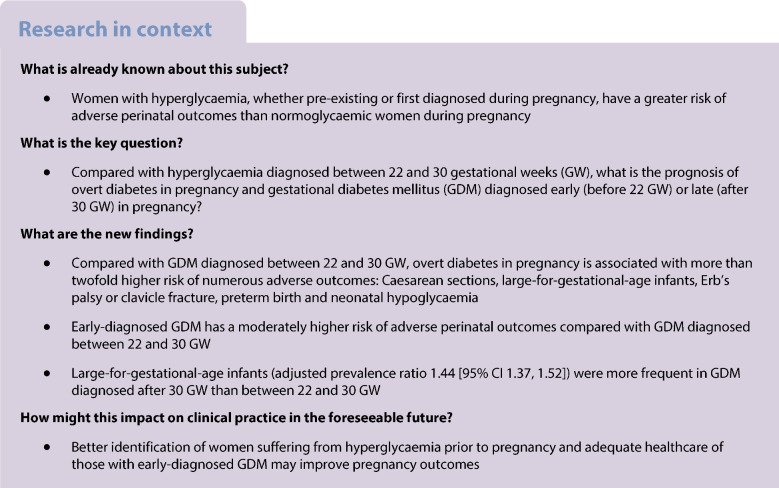


## Introduction

Hyperglycaemia is a common problem during pregnancy. In the Hyperglycemia and Adverse Pregnancy Outcome (HAPO) Study, which included 15 international participating centres, it affected 9.3% to 25.5% of the study population [1]. Both overt diabetes in pregnancy and gestational diabetes mellitus (GDM) contribute to hyperglycaemia in pregnancy [2, 3] and, despite treatment, they are associated with a higher risk of adverse outcomes [4].

The ‘overt diabetes in pregnancy’ category was introduced by the International Association of Diabetes Pregnancy Study Groups (IADPSG) to distinguish women who had unknown diabetes prior to pregnancy from those with GDM [3]. Overt diabetes in pregnancy has a worse prognosis than other types of hyperglycaemia in pregnancy [5, 6].

The French National College of Obstetricians and Gynaecologists and the French-speaking Society of Diabetes jointly propose risk-based screening for hyperglycaemia in pregnancy in line with the IADPSG recommendations [7]. First trimester screening among women at risk facilitates early identification and treatment [2, 3, 8]. In the case of normal laboratory test results, a 75 g OGTT is recommended between 24 and 28 gestational weeks (GW). GDM screening may also occur outside the screening schedule if ultrasound examinations identify macrosomia or polyhydramnios later in pregnancy [7, 9, 10].

Insulin resistance physiologically increases after 24 GW, but, in women with early-diagnosed GDM, it can develop earlier in their pregnancy with heightened resistance [11, 12]. Early-diagnosed GDM shares similar risk factors with impaired fasting glucose, impaired glucose tolerance and type 2 diabetes and is associated with a higher incidence of type 2 diabetes mellitus after delivery [11, 13].

As the prognostic value of early- and late-diagnosed GDM remains unclear [9–11, 13, 14], we conducted a large-scale national observational study using data from the French National Health Data System (Système National des Données de Santé, SNDS). The aims of the study were: (1) to estimate the proportion of each subtype of hyperglycaemia in pregnancy among all hyperglycaemic pregnant women in France in 2018; and (2) to compare the prognoses of those with normoglycaemic status, early-diagnosed GDM, late-diagnosed GDM and overt diabetes with women diagnosed with GDM between 22 and 30 GW (GDM_22–30_).

## Methods

### Data sources

The SNDS contains data from the French hospital discharge database and the French National Health Insurance information system. The hospital discharge database provides information on all hospital stays, while the National Health Insurance information system provides a record of all reimbursements made by the French National Health Insurance for individual out-of-pocket healthcare spending (e.g. outpatient prescriptions, outpatient medical visits). Information relating to the same patient can be matched using her or his unique anonymised identification number to create a specific database for a given study population.

The SNDS also includes socioeconomic data such as age and beneficiaries of French universal complementary medical coverage (couverture médicale universelle complémentaire, CMU-C) or state medical aid (aide médicale de l’État, AME), which are means-tested benefits granted for 1 year. AME is for undocumented migrants, whereas CMU-C is intended more broadly for low-income individuals. In 2018, for instance, the annual income limit for CMU-C or AME was €8810 for a single person.

The SNDS does not contain race or ethnicity data. In France, it is prohibited to analyse personal data that reveal racial or ethnic origins (law no. 78-17 of 6 January 1978). There are exceptions, but these do not apply to the SNDS.

### Study population

**Fig. 1:**
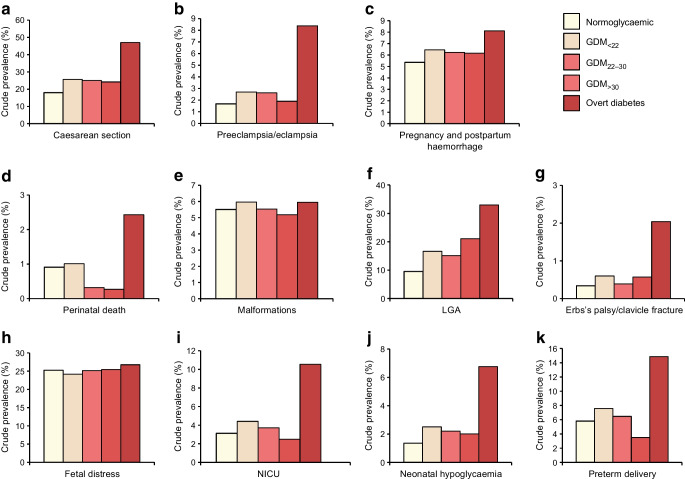
Crude rates of maternal and neonatal outcomes for the study according to maternal glycaemic status (*n*=695,912) (France, 2018). All overall *p* values were <0.0001, except for malformations, where the *p* value was 0.008

All deliveries in public and private maternity hospitals in France are recorded in the SNDS database. Our study included deliveries occurring in 2018. The SNDS does not record home births or deliveries in birthing centres that did not have subsequent in-hospital postpartum care. However, these account for less than 1% of deliveries in France [15]. From these records, we excluded deliveries on account of data link issues, deliveries with missing data or poor data quality and deliveries outside our study population (i.e. multiple births, women residing abroad or in Mayotte, second delivery in the year and women with pre-existing diabetes as described in a previous publication [4]) (electronic supplementary material [ESM] Fig. 1).

### Data

ICD-10 (https://icd.who.int/browse10/2019/en.) codes, procedure codes and other nomenclature codes used to generate the variables shown below are listed in ESM Table 1.

#### Hyperglycaemia in pregnancy

Women with at least one reimbursement for insulin during pregnancy, at least two reimbursements of glucose strips (≥200 strips) or women with a hospital diagnosis of diabetes either during pregnancy or at delivery [4] were identified as having hyperglycaemia in pregnancy.

In France (see ESM Fig. 2), GDM screening is recommended when at least one of the following criteria is met: maternal age ≥35 years; BMI ≥25 kg/m^2^; history of diabetes in a first-degree relative; personal history of hyperglycaemia in previous pregnancies; and previous delivery of a large-for-gestational-age (LGA) infant [7]. For these women, fasting plasma glucose (FPG) measurement is recommended during the first trimester and early-diagnosed GDM is defined as FPG level ≥5.1 mmol/l. In the case of normal laboratory test results, an OGTT is recommended between 24 and 28 GW, where hyperglycaemia is diagnosed when FPG is ≥5.1 mmol/l and/or the 1 h plasma glucose level is ≥10 mmol/l and/or the 2 h plasma glucose level is ≥8.5 mmol/l. Finally, screening with OGTT is indicated after 28 GW in the case of ultrasound evidence of macrosomia or polyhydramnios in women not diagnosed with hyperglycaemia earlier in pregnancy [7, 8] (ESM Fig. 2).

Women were classified as having overt diabetes in pregnancy in our study if they were identified as hyperglycaemic for the first time during the index pregnancy and continued to receive insulin or had a reimbursement for oral glucose-lowering agents at least three times in the following year. This group corresponded to women who probably had undiagnosed pregestational diabetes and, as a consequence, required hypoglycaemic agents after delivery. The other pregnant women with hyperglycaemia in the study population, presumably due to GDM, were subsequently categorised according to the timing of diagnosis.

Exploratory analyses of the distribution of the timings of OGTT in the present study suggested that OGTT appointments normally intended for 24–28 GW often took place slightly earlier or later, possibly at the discretion of the physician or because the timing was more convenient for patients (data not shown). Accordingly, we extended the screening period by adding 2 weeks before and after the recommended period. Women receiving an OGTT between 22 and 30 GW were therefore defined as being screened within the recommended screening window according to the French guidelines. This group was categorised as GDM_22–30_ (reference group). We then categorised all women with GDM according to their screening date into the two other subtypes of GDM: <22 GW (GDM_<22_) and >30 GW (GDM_>30_).

French recommendations state that women with hyperglycaemia in pregnancy should be referred immediately to healthcare professionals for tailored dietary advice and should commence blood glucose self-monitoring. Insulin treatment is prescribed when the preprandial glucose level or 2 h postprandial glucose level exceeds 5.3 or 6.6 mmol/l, respectively [7]. Oral hypoglycaemic agents are not prescribed during pregnancy in France. Mothers and infants are subsequently followed by obstetricians, midwives and paediatricians [7].

The dates of conception and last menstrual period were estimated using the gestational age at delivery recorded in the SNDS database. We defined the date of diagnosis of hyperglycaemia as the date of the last glycaemic screening assessment before the first healthcare prescription for test strips or the first insulin delivery or the first hospitalisation with a diagnosis of diabetes. The time between diagnosis and initiation of medical care was defined as the length of time between the date of diagnosis and the date of the first reimbursement of test strips or hospitalisation for diabetes. The time between GDM diagnosis and insulin treatment was defined as the length of time between the date of diagnosis and the date of the first insulin delivery.

#### Maternal outcomes

Obstetric outcomes included Caesarean sections, preeclampsia or eclampsia, and antepartum, intrapartum and postpartum haemorrhages during the delivery stay (ESM Table 1).

#### Neonatal outcomes

Neonatal outcomes were perinatal death (including stillbirth and death within the first 7 days of life), congenital malformations, LGA infant (birthweight >90th percentile for a given gestational age [16]), Erb’s palsy or clavicle fracture (for vaginal deliveries only), fetal distress, admission to the neonatal intensive care unit (NICU), neonatal hypoglycaemia and preterm delivery (birth at <37 GW) (ESM Table 1).

### Statistical analyses

We described the characteristics of women and the prevalence of pregnancy outcomes according to glycaemic status. We tested differences using ANOVA and χ^2^ tests.

Multilevel Poisson regression models with robust variance were used to estimate the adjusted prevalence ratio (aPR) and 95% CI, with hospital and mother levels to take into account that mothers who gave birth in the same hospital were not independent of each other. GDM_22–30_ was considered the reference group to calculate the prevalence ratios. To take into account the multiplicity of tests, an association was considered statistically significant according to the Holm–Bonferroni procedure, with a family-wise error rate at 5% and 84 tests (i.e. four comparisons in 21 models: ten outcomes declined for ≥31 GW and for ≥37 GW and one outcome declined for ≥31 GW only) [17, 18]. For all outcomes, the prevalence ratios were adjusted for maternal age (as a continuous variable), socioeconomic status and gestational age (as a continuous variable), except for preterm birth where the prevalence ratios were adjusted for maternal age and socioeconomic status.

Attempts were made to minimise immortal time bias during analysis [4, 19]. Immortal time refers to a period of follow-up during which the outcome of interest (GDM diagnosis and pregnancy outcomes) cannot occur by study design. A large proportion of women with GDM are diagnosed between 24 and 28 GW using the OGTT. These pregnancies must thus ‘survive’ until 24–28 GW in order to be screened for GDM. Restricting the analyses to deliveries at ≥31 GW reduces the potential for immortal time bias by eliminating differences in the follow-up period between women with and without GDM [19]. Therefore, we performed analyses limited to: (1) all deliveries at ≥31 GW (i.e. threshold to define GDM_>30_); and (2) all deliveries at ≥37 GW (i.e. preterm delivery was not possible in this subgroup).

All statistical analyses were performed with SAS Enterprise Guide (version 7.1, SAS Institute, Cary, NC, USA).

## Results

A total of 750,554 deliveries were recorded in the SNDS in France in 2018. After exclusions, our study population was 695,912 women for the descriptive analyses (see ESM Fig. 1 for the study flowchart). Multivariable analyses were thus performed on 688,627 and 654,902 dyads when restricting the population to births at ≥31 GW and ≥37 GW, respectively.

### Characteristics of the study population

Overall, 611,207 (87.8%) women in the study population were not diagnosed with hyperglycaemia in pregnancy: 11.2% did not receive any glycaemic assessment during pregnancy. Among the study population, 84,705 women had hyperglycaemia in pregnancy (12.2%), with the following distribution: GDM_<22_, 36.8%; GDM_22–30_, 52.4%; GDM_>30_, 10.4%; and overt diabetes first diagnosed during pregnancy, 0.4%.

The study population characteristics according to glycaemic status are shown in Table 1. Women with overt diabetes were older and were more likely to have CMU-C or AME coverage (i.e. low socioeconomic status) and to be hospitalised during pregnancy. Women with GDM_>30_ were less likely to be treated with insulin and to be hospitalised, while they were more likely to have had a short delay between diagnosis and care. Overall, 86.8% of women with GDM_>30_ had been screened before 22 GW and 92.4% before 31 GW.

**Table 1:** Characteristics of women according to glycaemic status during pregnancy *N*=695,912 (France, 2018)

Characteristic	No hyperglycaemia in pregnancy *n*=611,207 (87.8%)	GDM_<22_ *n*=31,134 (4.5%)	GDM_22–30_ *n*=44,412 (6.4%)	GDM_>30_ *n*=8789 (1.3%)	Overt diabetes first diagnosed during pregnancy *n*=370 (0.1%)
Mean maternal age	30.1 (5.3)	32.3 (5.3)	32.3 (5.3)	31.6 (5.4)	33.6 (5.6)
Benefitting from CMU-C or AME due to low socioeconomic status	22.3	27.0	23.3	28.8	40.8
Gestational age at diagnosis (GW)	NA	11.0 (4.6)	25.8 (2.1)	33.6 (2.3)	15.0 (8.5)
Time between diagnosis and first care (weeks)	NA	6.9 (7.1)	3.8 (3.7)	2.4 (2.2)	3.9 (4.7)
Insulin treatment	NA	36.5	23.1	10.1	83.0
Time between diagnosis and insulin treatment^a^ (weeks)	NA	11.0 (7.0)	5.8 (2.8)	2.9 (1.6)	5.0 (4.5)
Hospital stay for diabetes during pregnancy	NA	6.3	5.0	3.7	34.9

Data are mean (SD) or %

^a^Among insulin-treated women

All overall *p* values were <0.0001

NA, not applicable

### Prevalence of outcomes during pregnancy according to glycaemic status

Figure 1 shows that the crude prevalence of maternal and neonatal outcomes differed according to the women’s glycaemic status. For most of these events, women with overt diabetes in pregnancy had the highest prevalence of complications followed by women with GDM_<22_. The risk of fetal distress increased across all subtypes of hyperglycaemia.

### aPR of maternal and neonatal outcomes

**Fig. 2:**
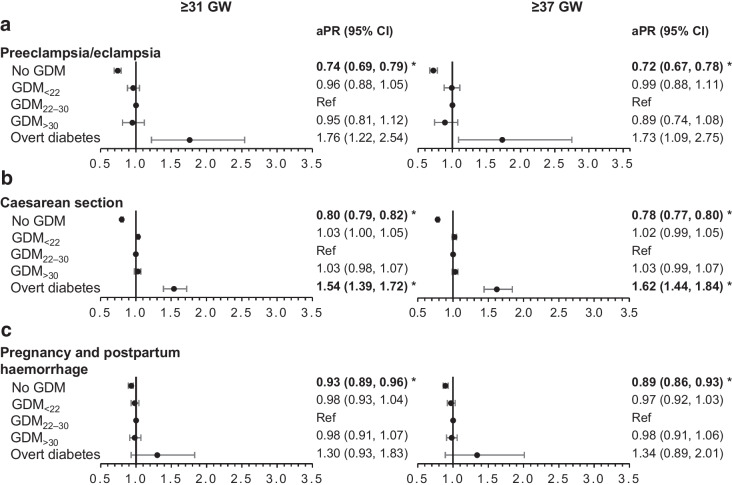
aPR of maternal outcomes among deliveries occurring at ≥31 GW (*n*=688,627 deliveries) or ≥37 GW (*n*=654,902) according to maternal glycaemic status (France, 2018). *p values statistically significant after Holm–Bonferroni adjustment. For each perinatal outcome, two forest plots are presented: one for deliveries at ≥31 GW (left) and one for deliveries at ≥37 GW (right). Ref, reference

**Fig. 3:**
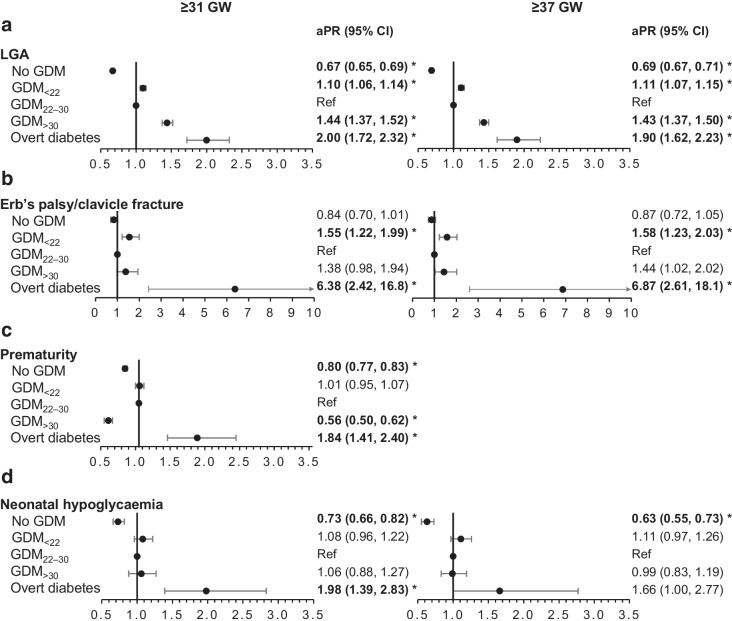
aPR of neonatal outcomes (LGA, Erb’s palsy/clavicle fracture, prematurity, neonatal hypoglycaemia) among deliveries occurring at ≥31 GW (*n*=688,627 deliveries) or ≥37 GW (*n*=654,902) according to maternal glycaemic status (France, 2018). *p values statistically significant after Holm–Bonferroni adjustment. For each perinatal outcome (except for prematurity), two forest plots are presented: one for deliveries at ≥31 GW (left) and one for deliveries at ≥37 GW (right). Ref, reference

**Fig. 4:**
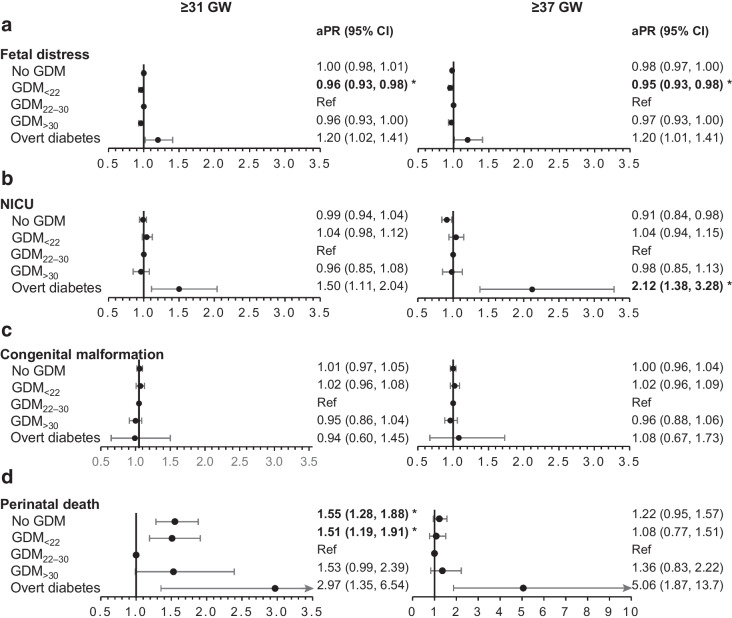
aPR of neonatal outcomes (fetal distress, NICU, congenital malformation, perinatal death) among deliveries occurring at ≥31 GW (*n*=688,627 deliveries) or ≥37 GW (*n*=654,902) according to maternal glycaemic status (France, 2018). *p values statistically significant after Holm–Bonferroni adjustment. For each perinatal outcome, two forest plots are presented: one for deliveries at ≥31 GW (left) and one for deliveries at ≥37 GW (right). Ref, reference

In Figs 2, 3, 4, we report the maternal and neonatal outcomes for the 688,627 women who gave birth at ≥31 GW and for the 654,902 women who gave birth at ≥37 GW. Women with GDM_22–30_ were the reference group in all our analyses. Overall, the results were similar in the two samples.

Women without hyperglycaemia in pregnancy were less likely to have preeclampsia or eclampsia, Caesarean section, and pregnancy and postpartum haemorrhage, while women with overt diabetes were more likely to have Caesarean section than those with GDM_22–30_ (Fig. 2).

Neonates born to women without hyperglycaemia in pregnancy were less likely to be LGA, be born preterm or have neonatal hypoglycaemia than those born to women with GDM_22–30_. However, they had a higher risk of perinatal death (deliveries at ≥31 GW only) (Figs 3, 4).

Neonates born to women with GDM_<22_ were more likely to be LGA and to have Erb’s palsy or clavicle fracture than those born to women with GDM_22–30_. They also had a higher risk of perinatal death (deliveries at ≥31 GW only).

Neonates born to women with GDM_>30_ were more likely to be LGA and were less likely to be born preterm than those born to women with GDM_22–30_.

Neonates born to women with overt diabetes were more prone to be LGA and preterm, to experience Erb’s palsy or clavicle fracture and to have neonatal hypoglycaemia. Neonates born to women with overt diabetes at term had a greater risk of NICU admission. Although the estimation of the aPR of perinatal death was five times higher (5.06 [1.87, 13.7]) for women with overt diabetes, these results were non-significant after Holm–Bonferroni adjustment.

## Discussion

### Principal findings

First, our results from a large study population show that compared with GDM_22–30_, overt diabetes was associated with more than twofold higher risk of numerous adverse outcomes. Second, some prognoses related to hyperglycaemia in pregnancy differed according to the timing of diagnosis. GDM_<22_, which represented 37% of all those diagnosed with hyperglycaemia in our study, was associated with a higher risk of adverse neonatal outcomes compared with GDM_22–30_. These results highlight the need to better identify women with hyperglycaemia in early pregnancy. Finally, GDM_>30_ was associated with more LGA neonates than GDM_22–30_.

Unsurprisingly, the risk of adverse maternal and neonatal outcomes was higher for women with any subtype of hyperglycaemia in pregnancy, especially in the GDM_<22_ group, than for normoglycaemic women. This suggests that healthcare provision can still be improved, especially for women who are diagnosed early or late in pregnancy.

### Strengths and limitations of the study

This nationwide study has important strengths. First, we had access to data from approximately 700,000 deliveries in France in 2018 and we were able to link maternal and neonatal data for 98.6% of them. Furthermore, to our knowledge, this is the first study using a large medico-administrative database to explore the consequences of specific subtypes of hyperglycaemia in pregnancy. Thanks to the use of a large-scale quasi-exhaustive database, we were able to include a sufficiently large number of women in each subtype of hyperglycaemia in pregnancy, including overt diabetes in pregnancy, to evaluate multiple outcomes. Second, the use of restricted analysis to account for immortal time bias increased the robustness of our study. In theory, maternal and fetal consequences of severe preeclampsia and intrauterine growth restriction that occur before 31 GW could lead to medically indicated preterm delivery, even before women belonging to the GDM_>30_ category were diagnosed [20]. Furthermore, immortal bias can explain the low rates of premature neonates born to women with GDM_>30_ as well as the higher risk of perinatal death of normoglycaemic women and women with GDM_<22_ for deliveries at ≥31 GW and not for deliveries at ≥37 GW, as previously reported [4]. One drawback of this approach of limiting the analyses to deliveries at ≥31 GW and ≥37 GW is that the results restricted to deliveries at ≥31 GW may not be generalisable to the entire population of pregnant women. Additionally, when focusing on deliveries at ≥37 GW, the association between overt diabetes and perinatal mortality risk may be affected by the reduced power caused by the low frequency of events and the Holm–Bonferroni procedure.

This study also has some limitations. First, the algorithm for the variable ‘overt diabetes in pregnancy’ includes only women who were treated pharmacologically for diabetes in the year following this delivery. Accordingly, those who managed their diabetes using only dietary measures were excluded from this group, which would have certainly underestimated the prevalence of overt diabetes in pregnancy based on its usual definition (FPG and/or 2 h post OGTT glucose and/or HbA_1c_ defining diabetes outside pregnancy) [5, 21–24]. However, our definition is more specific than the usual definition in terms of identifying women with unknown pregestational diabetes. As shown in the literature, up to 40% of women with overt diabetes (again, according to its usual definition) return to normal glucose tolerance at 6–8 weeks postpartum [22, 24]. One should note that in our study, women with overt diabetes were more often socioeconomically disadvantaged and potentially more likely to have undiagnosed diabetes prior to pregnancy. Second, we did not have information about glycaemic control. Poor glycaemic control is an important risk factor for outcomes such as LGA infants, clavicle fractures and perinatal mortality. Third, although we adjusted for socioeconomic status, maternal age and gestational age, other confounding factors at the patient or hospital level may have affected the relationship between the timing of GDM diagnosis and outcomes. However, we performed a multilevel model to take into account the hospital level. We lacked data on other comorbidities such as maternal overweightness and obesity, which are risk factors for both hyperglycaemia in pregnancy and some of the studied outcomes [25, 26]. Finally, 10% of the population did not have glycaemic screening during pregnancy. Some in this subgroup may have had undiagnosed hyperglycaemia in pregnancy. However, the impact on the comparisons between the different GDM categories would have been limited, even though the normoglycaemic group may have included some undetected GDM_22–30_ cases (hence, missing from the GDM_22–30_ category) with a different risk for adverse events compared with the detected GDM_22–30_ cases (e.g. undetected cases may have relatively mild GDM). Moreover, GDM cases can be identified late in pregnancy in cases of polyhydramnios or macrosomia diagnosed on ultrasound (GDM_>30_ in the present study) [7, 9, 10].

### Interpretation

In the present study, we observed an increase in the rate of hyperglycaemia in pregnancy in France between 2012 (7.2%) [4] and 2018 (12.2% of our cohort, 13.5% of all pregnant women). The increases in overweightness in pregnant women in France [27] and maternal age, which are both risk factors for GDM, contribute to this rise. A few other studies, though not all, have shown an increase in hyperglycaemia in pregnancy in recent years [27, 28]. The observed increase between 2012 and 2018 in France might also be partly explained by the higher levels of early screening uptake in accordance with the French recommendations published in late 2010 [7]. In the USA, implementing early screening nearly doubled the incidence of hyperglycaemia in pregnancy compared with the previous standard two-step approach [29].

In contexts where screening for GDM_<22_ is implemented, it has been shown to be very common. FPG level ≥5.0 mmol/l was found in 11.9% of pregnant women during the first trimester of pregnancy (on average at 9 GW) in Israel [30], while 11.4% had FPG >5.1 mmol/l at the first prenatal visit in China [31] and 7.2% in Italy [32]. Interestingly, GDM_<22_ accounted for 37% of women with hyperglycaemia in pregnancy in our study, which is consistent with previous findings in France [33] and internationally [34]. To our knowledge, this is the first study to explore the share of GDM_>30_ in women with hyperglycaemia in pregnancy.

Normoglycaemic women have a lower risk of adverse outcomes compared with women with GDM_22–30_ receiving care. Accordingly, Li et al recently found residual risk associations between hyperglycaemia and adverse GDM-related outcomes after a glycaemia-controlling intervention [35]. In fact, the risk of LGA infants is reported to be globally similar to that in women without GDM but with high normal glucose values [36].

In our study, compared with women with GDM_22–30_, women with overt diabetes were more likely to have Caesarean sections, while their neonates were approximately two times more likely to be LGA, to have Erb’s palsy or clavicle fracture, to be premature, to experience neonatal hypoglycaemia and to be admitted to the NICU. For Erb’s palsy or clavicle fracture, the results for babies born at ≥37 GW were the most relevant in clinical terms, although they did not differ when considering babies born at ≥31 GW. Other studies showed a higher rate of LGA infants [22, 24], shoulder dystocia [24], Caesarean section [6, 22], neonatal hypoglycaemia [6, 24] and pregnancy-induced hypertension [6, 23] in women with overt diabetes. Although the risk of congenital malformations was reported to be higher in overt diabetes in Italy [21] and France [6], we did not observe any such increase. It is possible that HbA_1c_ levels might not have been high enough at the time of conception to induce malformations in our series. Furthermore, the effect of hyperglycaemia on congenital anomalies may also be more difficult to show, because it might result in miscarriage or pregnancy termination before 22 GW. These outcomes were not included in our study, which instead focused on maternal and perinatal outcomes after 22 GW.

A meta-analysis comparing pregnancy outcomes between early-diagnosed and late-diagnosed GDM [12] showed that women in the first group had a significantly higher likelihood of perinatal mortality risk (RR 3.58 [95% CI 1.91, 6.71]), neonatal hypoglycaemia (RR 1.61 [1.02, 2.55]) and insulin use (RR 1.71 [1.45, 2.03]). However, no difference was observed between the two groups in terms of mean birthweight, LGA and small-for-gestational-age infants. Previous cohort-based studies highlighted that women with early-diagnosed GDM in high-income countries also had a significantly higher likelihood of delivering infant neonates requiring NICU admission (RR 1.12 [95% CI 1.0, 1.22]). Our results may differ because these studies used OGTT or HbA_1c_ to define early-diagnosed GDM [14, 37] as opposed to FPG alone, which is the recommendation in France. Additionally, other countries use higher thresholds of FPG to define early-diagnosed GDM than the threshold used in France [38, 39], while the diagnostic criteria for early-diagnosed GDM are controversial [40]. However, poor prognosis following elevated FPG levels during the first trimester was previously reported, including an increased risk of macrosomia [30, 41, 42] and preeclampsia [30]. Applying a risk-based screening model based on IADPSG criteria to a large multi-ethnic cohort in Australia, women diagnosed at <24 GW and identified as having early-diagnosed GDM were also associated with having poorer pregnancy outcomes [43], including gestational hypertension in a recent French study [6]. Treatment for GDM before 20 GW can slightly reduce adverse neonatal outcomes [14].

Women may be referred for GDM screening after 30 GW when macrosomia or polyhydramnios is suspected. In our cohort, approximately 90% of women with GDM_>30_ were screened at least once before 30 GW. These women were probably retested following the diagnosis of macrosomia or polyhydramnios on ultrasound despite their previous screening results being normal [7, 9, 10]. The remaining women (10%) may have been diagnosed after 30 GW because of ultrasound abnormalities or because they did not follow the recommended screening schedule earlier in pregnancy if they had risk factors for hyperglycaemia in pregnancy. Interestingly, the GDM_>30_ group had the lowest percentage of women requiring insulin (10%). This finding warrants further studies to understand whether the lower rate of insulin prescription could explain the higher rate of LGA infants in women with GDM_>30_ compared with those with GDM_22–30_, as previously reported [9].

### Conclusion and perspectives

To conclude, hyperglycaemia in pregnancy remains associated with poor pregnancy outcomes despite early testing and current best practices regarding immediate care and treatment. Overt diabetes in pregnancy is associated with a higher risk of adverse maternal and neonatal outcomes. Our study suggests that screening at-risk populations for hyperglycaemia in the preconception period is important. Screening can also identify women with prediabetes who are prone to developing hyperglycaemia early in pregnancy. Our findings also show that women with early-diagnosed and late-diagnosed GDM had a higher risk of adverse outcomes. This suggests that diagnostic and management pathways can still be improved [11, 12, 44]. Large-scale prospective clinical studies focusing on the different subtypes of hyperglycaemia in pregnancy according to the time of onset and diagnosis as well as on overt diabetes in pregnancy are necessary. More data on phenotypes, comorbidities and glycaemic control are required to produce conclusive evidence.

### Supplementary Information

Below is the link to the electronic supplementary material.

Supplementary file1 (PDF 272 KB)

## Data Availability

Santé publique France (the national public health agency) has permanent access to the SNDS database and is committed to publishing its findings. However, we cannot publicly provide data extracted from the French National Health Insurance information database due to restrictions set by our ethics committees, unless an agreement between all parties has been reached.

## Abbreviations

AME: Aide médicale de l’État (state medical aid for undocumented migrants)
aPR: Adjusted prevalence ratio
CMU-C: Couverture médicale universelle complémentaire (complementary universal medical coverage)
FPG: Fasting plasma glucose
GDM: Gestational diabetes mellitus
GDM_<22_: Gestational diabetes mellitus diagnosed before 22 gestational weeks
GDM_22–30_: Gestational diabetes mellitus diagnosed between 22 and 30 gestational weeks
GDM_>30_: Gestational diabetes mellitus diagnosed after 30 gestational weeks
GW: Gestational weeks
IADPSG: International Association of Diabetes Pregnancy Study Group
LGA: Large-for-gestational-age
NICU: Neonatal intensive care unit
SNDS: Système National des Données de Santé (French National Health Data System)
