# Supplementary material for: Maternal and neonatal outcomes according to the timing of diagnosis of hyperglycaemia in pregnancy: a nationwide cross-sectional study of 695,912 deliveries in France in 2018
Source: Diabetologia. 2024 Jan 5;67(3):516–27. doi: 10.1007/s00125-023-06066-4 (PMC10844424; doi:10.1007/s00125-023-06066-4)
Supplement: Supplementary file 1 — Supplementary file1 (PDF 272 KB) [file 125_2023_6066_MOESM1_ESM.pdf]

ESM table 1: codes used in the SNDS database to create variables needed for the study

**Delivery stays**	**ICD-10: Z37**
Pregnancy start date	= Date of delivery – (gestational age in completed weeks * 7 + 14)
**Diabetes status**	
Hospitalization with diabetes	ICD-10: E10, E11, E12, E13, E14, E220, G632, H360, O24 or R739
Insulin	ATC: A10 (except A10BX06) with the mention "Insulin"
Oral glucose-lowering agents	ATC: A10 (except A10BX06) without the mention "Insulin"
Glucose strips	LPP: 1173487, 1136894,1186722,1180441 or 1187408
Glucose assay	NABM: 412, 413 or 552
**Maternal outcomes**	
Caesarean section	CCAM : JQGA002, JQGA003, JQGA004 or JQGA005
Preeclampsia or eclampsia	ICD-10 : O14 or 015
Antepartum, intrapartum and postpartum haemorrhages	ICD-10 : O441, O67, O72 or O902
**Live birth or stillbirth stays**	**ICD-10 : Z38 or P95**
**Neonatal outcomes**	
Perinatal death	ICD-10 : P95 or death within the first 7 days of life
Congenital malformations	ICD-10 : Q00-Q99
Erb’s palsy or clavicle fracture	ICD-10 : P140, P141, P142, P143 or P134 for vaginal deliveries only
Fetal distress	ICD-10 : O68 (in the delivery discharge data)
Neonatal hypoglycaemia	ICD-10 : P704

Abbreviations:

ICD-10: International Classification of Diseases, 10th edition

ATC: Anatomical Therapeutic Chemical classification

LPP (*Liste des Produits et des Prestations)*: French nomenclature for medical devices

NABM (*Nomenclature des actes de biologie médicale)*: French nomenclature for medical biology procedures

CCAM (C*lassification Commune des Actes Médicaux)*: French nomenclature for medical procedures

ESM Fig. 1: study flowchart

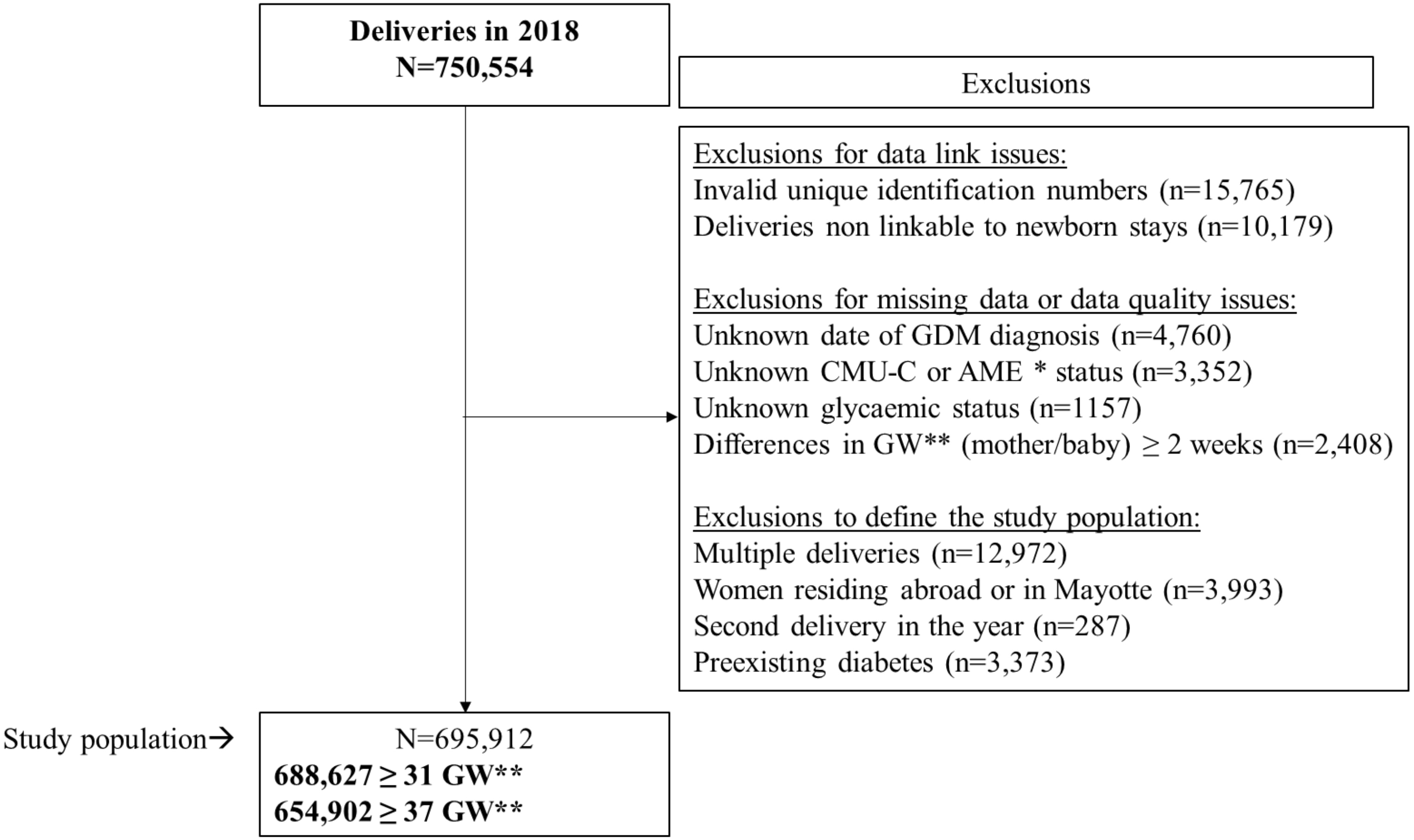

Legend:

* CMU-C: complementary universal medical coverage

* AME: state medical aid for undocumented migrants

** GW: gestational week

ESM Fig. 2: French recommendations for screening for hyperglycaemia in pregnancy

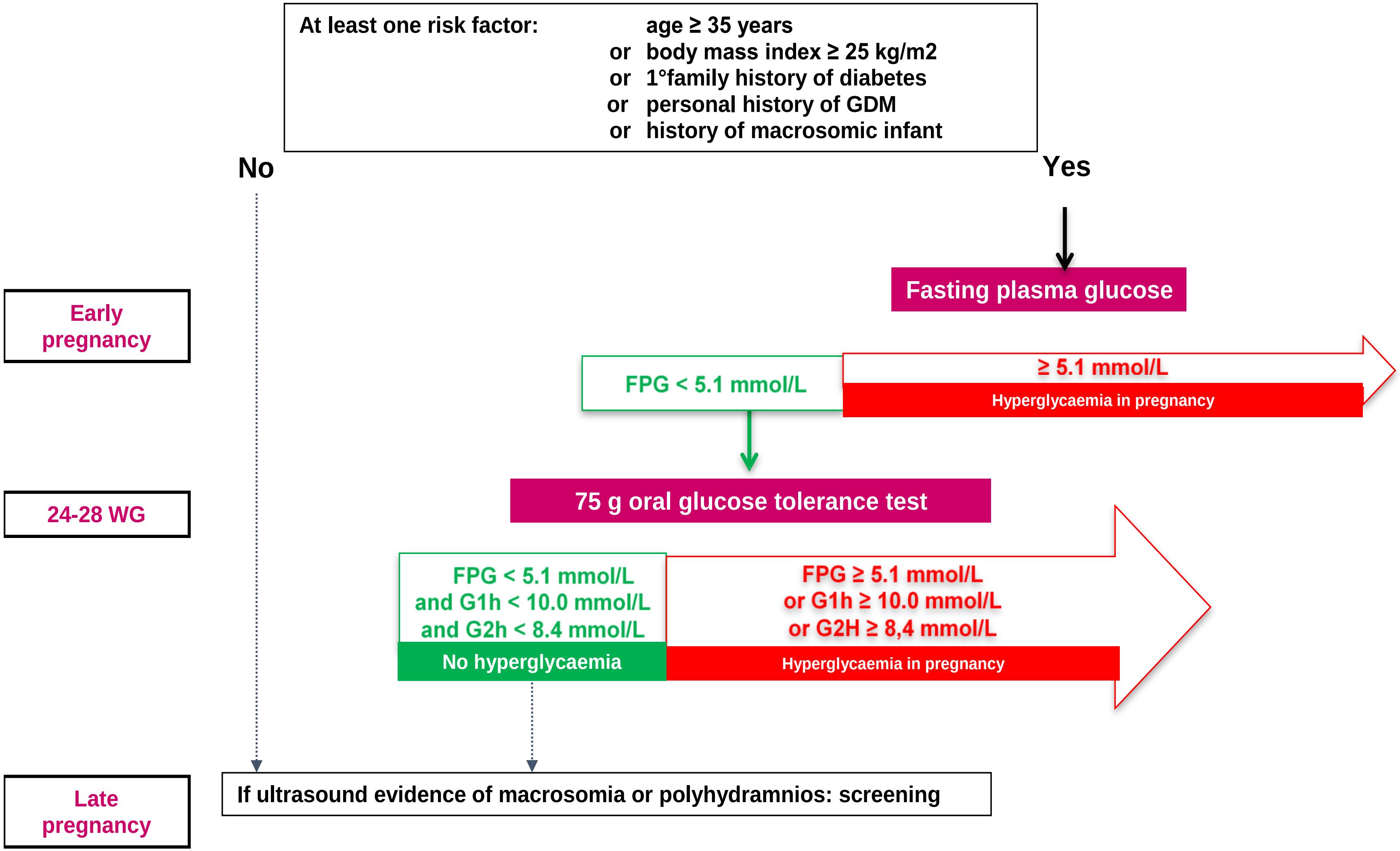

Legend:

This algorithm shows current French recommendations

FPG: fasting plasma glucose; G1h and G2h: plasma glucose value 1 and 2 hours after 75g oral glucose tolerance test, respectively;

WG: weeks of gestation

Plasma glucose value: 5.1 mmol/L = 92 mg/dL ; 8.5 mmol/L = 153 mg/dL ; 10.0 mmol/L = 180 mg/dL

Reference: Gestational diabetes. Summary of expert consensus. Diabetes Metab 2010;36:695-699
